# Supplementary material for: Content-rich biological network constructed by mining PubMed abstracts
Source: BMC Bioinformatics. 2004 Oct 8;5:147. doi: 10.1186/1471-2105-5-147 (PMC528731; doi:10.1186/1471-2105-5-147)
Supplement: Additional File 2 — The original results of the above study (non-essential files are deleted to keep the file size under the limit set by BMC bioinformatics). [file 1471-2105-5-147-S2.bz2 › chilibotAdditionalFile2/dip05/733ID11983079E18091/html/FDX_ISCS.html]

 


 **FDX** and **ISCS** 
  
Found 11 abstracts in PubMed, retrieved 05.  
 

 What does Google say? 
 PDF only 
| .edu only 

---

**Interactive relationship** (e.g. stimulation, inhibition, etc)

**Neutral relationship**- In addition, we have identified previously unreported interactions between  **IscS**  and  **Fdx** ,  **IscS**  and ORF3, IscA and HscA, and HscA and  **Fdx** .  Ref: 11983079 J Biochem Tokyo , 2002
- Here we show that cysteine desulfurase  [ **ISCS** ]  encoded by the gene  **iscS**  of E. coli can directly convert the ferredoxin  [ **FDX** ]  dinitrosyl iron complex to the ferredoxin 2Fe 2S  [ **FDX** ]  cluster in the presence of L cysteine in vitro.  Ref: 11825893 J Biol Chem, 2002
- The results suggest that cysteine desulfurase  [ **ISCS** ]   **IscS**  together with L cysteine can efficiently repair the nitric oxide modified ferredoxin 2Fe 2S  [ **FDX** ]  cluster.  Ref: 11825893 J Biol Chem, 2002

**Non-interactive relationship** (e.g. studied together, co-existance, homology, etc.)

- Thus, the sequential association and dissociation among the  **IscS** , IscU, IscA, HscB, HscA,  **Fdx** , and ORF3 proteins may be a critical process in the assembly of Fe S clusters.  Ref: 11983079 J Biochem Tokyo , 2002
- Repair of nitric oxide modified ferredoxin 2Fe 2S  [ **FDX** ]  cluster by cysteine desulfurase  [ **ISCS** ]   **IscS** .  Ref: 11825893 J Biol Chem, 2002
